# Sexual experiences of cancer survivors: A qualitative study in Jordan

**DOI:** 10.1371/journal.pone.0271264

**Published:** 2022-08-31

**Authors:** Muhammad Al-haddad, Ibrahim M. Alananzeh, Heidi Green, Albara Alomari, Ritin Fernandez

**Affiliations:** 1 Al Khalidi Hospital, Amman, Jordan; 2 School of Humanities, Social Sciences and Health, University of Wollongong in Dubai, Dubai, United Arab Emirates; 3 School of Nursing and Midwifery, University of Wollongong, Wollongong, Australia; 4 Centre for Research in Nursing and Health, George Hospital, Centre for Evidence Based Initiatives in Health Care, Sydney, Australia; 5 Hamad Medical Corporation, Doha, Qatar; University of Perugia: Universita degli Studi di Perugia, ITALY

## Abstract

**Background:**

In Jordan, cancer is the second leading cause of death after cardiac disease. The impact of cancer on sexual relationships is a taboo subject and hence, such issues are underreported research among Jordanian people examining unmet needs.

**Aim:**

To examine the experiences and preferences of Jordanian cancer survivors related to communication regarding their sexual needs.

**Methods:**

Qualitative study conducted between May and June 2020 using semi-structured face-to-face interviews using the snow-balling approach for recruitment. Participants were recruited until data saturation was obtained and data were analyzed using qualitative thematic analysis.

**Results:**

Analysis of participants’ interview data identified three main categories: 1) The psychological impact of cancer of sexual relationships; 2) Physician support; 3) Variations in sexual life and sexual experiences.

**Conclusion:**

The study revealed that there is a substantial psychological impact of cancer on sexual relationships among Arab Jordanian cancer survivors. Feeling powerless, being labelled as ‘sexually disabled’, and lack of communication with physicians were emphasized by participants as a barrier to discuss sexual needs with their physicians. Lack of physicians support negatively impact Jordanian cancer survivors sexual experience and led to increased their sense of powerlessness and loss of control over sexual relationships.

**Implications for practice:**

Overall, the study indicated a crucial need for physicians to encourage patients to disclose their sexual experience to help them maintain their sexual and mental health while in the fight against cancer. The physicians, nurses and allied health professionals should be engaged in the conversation with patients by taking an active role in the discussion. Additionally, the family and partners of the patients should also be approached and engaged by health professionals in the discussion to address their needs or sexual issues.

## Introduction

Cancer is the second leading cause of death globally, accounting for an estimated 9.6 million deaths, or one in six deaths, in 2018 [1]. The five most common types of cancer reported worldwide [2] were lung, breast, colorectal, prostate, and stomach cancers. In Jordan, cancer remains a major illness and the second leading cause of death after cardiac diseases [3]. According to Jordan Ministry of Health reports, there were 4849 cases of cancer in Jordan in 2010, and this number has increased to 5999 in 2016. Breast, colorectal, lung, lymphoma, and leukaemia were the most common cancers [4]. Rapid population growth and continuous advancement in healthcare research and technology have increased the number of cancer survivors, placing a higher burden on healthcare providers to meet the emotional, social, and sexual needs of cancer survivors.

Sexual health is considered an essential part of patients’ overall quality of life, including those affected by cancer. Changes in sexual needs among cancer survivors have been attributed to the disease, the physical and emotional impact of cancer diagnosis and treatment, and a repositioning of themselves as patients, not sexual partners [5]. These sexual changes can have a significant impact on a cancer survivor’s self-esteem and mental health and can contribute to feelings of depression, anxiety, and other mental health issues [6].

Research on cancer care indicates that cancer survivors receive inadequate support relating to their sexual needs during their cancer journey [7], resulting in poor quality of life and high levels of depression as a result of unmet needs [8]. In Arab culture, talking about sexual needs within as well as outside the family is a taboo subject [9]. In a large study undertaken on Arab Australian and Arab Jordanian cancer survivors, more than 40% of the respondents reported that not finding staff to talk to was their greatest unmet need. More than 35% of the respondents indicated that their unmet needs included fear of uncertainty, having written information about care, and information on how to help themselves [7].

Cancer survivors have their own unique fight against the disease, in addition to its impact on their sexual and psychosocial lives. While there is extant literature on the supportive care needs among Arab cancer survivors and links those needs to their clinical, psychosocial, and sociodemographic characteristics [7]. Among Arab culture talking about bodies and sex in the Arab world can still be considered taboo. There’s even a dedicated Arabic word for it, “ayb”, leaving many people with nowhere to turn to for accurate information and help [10]. The little information available about the Jordanian cancer survivors is relevant to the cultural stigma and shame associated with cancer in general and around sexuality in particular [11]. Jordanian cancer survivors believe that not discussing sexuality with anyone is a way to maintain confidentiality and avoid the stigma that is associated with a cancer diagnosis and cancer treatment side effects [12].

There are limited studies on the sexual unmet needs in this population. Therefore, the aim of this study was to explore the sexual needs among Jordanian people affected by cancer using a qualitative study design.

This study will examine the experiences and preferences of Jordanian cancer survivors related to communication regarding their sexual needs. The study is unique in that it is the first to understand the sexual health needs of Jordanian people affected by cancer and connect these needs with their cultural beliefs and practices. Findings from this study may inform future health services for this community and address a significant knowledge gap about the sexual health needs of cancer survivors in the Middle East region.

## Methods Research design

Using a qualitative exploratory design, this study focused on Jordanian cancer survivors experience with cancer care providers, including nurses, in meeting information needs regarding sexuality. This study follows the consolidated criteria for reporting qualitative research (COREQ) 32-item checklist [13].

### Sampling and recruitment method

Adult Jordanian males, who were married cancer survivors aged 18 years or older and able to read and/or speak Arabic were invited to participate in the study. For the purpose of this study, we used the National Institutes of Health definition of cancer survivor to mean the period from the time of diagnosis until the end of life [14].

Prospective participants in the community, (seven married males), were directly recruited by the primary investigator (MA) who knew they had cancer through the word of mouth and snowballing. Participants were provided with written information that explained the study aim, eligibility criteria, and the consent process. Verbal consent to participate in the study was obtained. Participants were offered the opportunity to be interviewed via either telephone or face-to-face. Participants were interviewed at a mutually agreed time and location. Participants who agreed to be interviewed were asked to distribute study details to other Jordanian cancer survivors who they felt might also be interested in participating (snowball sampling).

### Study setting

The study was undertaken in the community in Amman, Jordan where Arabic is the primary language.

### Data collection

All participants agreed to semi-structured face to face interviews, which were conducted in June 2020. Interviews were conducted by the study primary investigator who is a male nurse with extensive experience in cancer nursing, fluent in Arabic and used the same dialect as the study participants.

Interviews were conducted using a semi-structured interview guide developed by the research team to explore survivors’ experiences relating to sexual needs. The main topic areas included: the importance of sexuality to cancer survivors, the impact of cancer and its treatment on their sexual life, information and support needs, healthcare providers addressing sexuality needs, and sexual partner engagement.

Whilst being able to speak Arabic made the interviewer linguistically competent, care was taken to not assume ’insider’ status because of the sensitivity of the subject for participants. Confidentiality was emphasized and a culturally sensitive approach was taken by the researcher being respectful of participants’ experiences and beliefs when talking about their sexual experience. Interviews were conducted until the research team determined data saturation had been achieved. Participants were interviewed individually in a location mutually agreed and private location. Interviews were audio-recorded and ranged from 29 to 43 minutes.

### Data analysis

All interviews were digitally recorded and transcribed verbatim, by a professional transcription service. The transcripts were not returned to participants for comments. The transcripts were anonymized by providing participants with pseudonyms and checked against the audio recordings to ensure the accuracy of the transcription. Initially, three authors (IA, MA, BA) analyzed the Arabic transcripts thematically using an inductive approach as described by Braun and Clark [15]. Thematic analysis involved six steps. The first step was familiarization with the data, whereby each author read the transcripts multiple times. Once familiarization occurred, quotes were classified and clustered into themes. The themes were revised and refined until consensus was reached among the three authors [15]. Once the themes were created in Arabic, these were then translated to English by co-authors (IA, MA). To ensure the rigour of the analysis three co-authors (RF, HG and IA) appraised the analysis process in the audit trail, confirming that all possible themes had been identified. To maintain confidentiality and privacy of participants’ identity, pseudonym names were used in the quotes.

### Ethical considerations

The study received ethics approval from the University of Wollongong approval number 2019/373. Participants were provided with verbal and written information about the study and were informed that participating in the study was voluntary. Participants were assured of confidentiality and anonymity about the information that they provided.

### Trustworthiness of qualitative data

All elements of trustworthiness, including credibility, dependability, and transferability were considered in this study [16]. The credibility of the data was enhanced through selecting a suitable research team, applying purposeful sampling and collecting data by a native Arab researcher who was fluent in Arabic. In addition, interviews were commenced by inviting the study participants to ’tell their story’, an approach which not only helps build rapport but shows respect for participants’ experiences [17]. In order to ensure dependability peer reviewing was undertaken to verify the findings and an audit trail was maintained. Finally, transferability was confirmed by assessing the applicability of these themes to other participants. Although the findings obtained were from male cancer survivors, some aspects could be relevant to women.

### Findings

Seven male participants aged between 42 and 62 years were interviewed. The participants were cancer survivors for a range of 1 month to 1 year. Cancer type and occupation varied for all participants. Participant’s educational level ranged from high school diploma to master’s degree. All participants were married and had children Table 1 provides the demographics of the participants.

**Table 1:** Demographics data of the participants.

	_Participant 1	Participant 2	Participant 3	Participant 4	Participant 5	Participant 6	Participant 7
**Age**	62	50	54	47	42	44	57
**Length of time of cancer**	1 month	6 months	2 months	6 months	5 months	3 months	1 year
**Type of cancer**	Prostate Cancer	Leukemia	Colon Cancer	Gastric Cancer	Pancreatic Cancer	Thyroid Cancer	Prostate Cancer
**Employment**	Army retiree	Construction worker	School teacher	Architecture	Bank Accountant	Internal Audits	Driver
**Education Level**	College Diploma	College Diploma	Masters	Bachelor	Master	Bachelor	High School Diploma
**Marital Status**	Married	Married	Married	Married	Married	Married	Married
**Children**	6	3	5	4	5	4	3

Three main themes emerged from the qualitative analysis: 1) The psychological impact of cancer of sexual relationships; 2) Physician support; 3) Variations in sexual life and sexual experiences. Table 2, provide a summary of the themes.

**Table 2:** Summary of the themes.

Themes	Subthemes
The psychological impact of cancer on sexual relationships	Shame associated with discussing sex life
	Feeling powerless
	Mental wellbeing and sexual desire
Need for a therapeutic/intimate communication	Need for information about the effects of cancer on sexual relationships
	Building a rapport is vital for openness
	It is a psychological issue, not the cancer
	The need for sexual counselling as part of the cancer care
Variations in sexual life and sexual experiences	Sources of information regarding the impacts of cancer on sex life
	Effects on the partner
	Sexual relationships prior to cancer

### 1 The psychological impact of cancer on sexual relationships

#### 1.1 Shame associated with discussing sex life

The cultural and societal norms of providing an insight into their marital relationships, including sex life, was described by the participants as a taboo topic. One participant stated *“In my opinion*, *the culture of the people*, *because this topics are mainly sensitive*, *sensitive and personal topics*, *so you find that they feel shy and sensitive to talk about it”* (Participant 5), thus preventing any conversation about sexual relationships. A strong feeling of shame is also highlighted by participants as a reason for not wanting to talk about sexual issues, suggesting *“The main reason for sure is shame*, *doctor*, *saying that this patient is sick and I want to treat him*, *and anything else (like sexual issues) in his life*, *it’s not my business”* (Participant 1). Issues regarding being labelled a defect by their doctor and that sexual issues being a private matter are barriers to discussing sexual intimacy, one participant said: *“In the end*, *the doctor comes to the patient and says defect (shame and stigma)*, *I do not speak about these topics*!*”* (Participant 2).

#### 1.2 Feeling powerless

A sense of powerlessness and loss of control over their sexual relationships and experiences was highlighted by participants in this study. One participant explained *“Now*, *after the disease*, *the erection is very weak and for a very short period”* (Participant 1), demonstrating the struggle to maintain a normal sex life. Another participant talked about *“The disease made me unable to practice my marital relationship (sex life) as it was in the first place”* (Participant 3), also discussing the changes in sexual experiences which has an impact on their mental status. Others felt as if cancer is a major barrier in their sexuality resulting in inability to continue their sexual relationships with their partners, “*I have a disability (I was unable to do it)*, *a deficit in the sense of distance*. *I mean*, *even if I want (to have sex) this relationship*, *I am really unable”* (Participant 3).

#### 1.3 Mental wellbeing and sexual desire

Sexual desire for many participants was affected by their altered mental status. One of the greatest obstacles to sexual desire is the individuals’ psychological wellbeing, *“Mentally you will be destroyed and your mental statues will be very bad*. *Thinking about the relationship (sexual relationship) was less in a different universe and I was in fear from the disease itself”* (Participant 4). The desire for continuing a sexual relationship with their partner after a cancer diagnosis was suggested to be related to their mental stability rather than physical ability to perform. One participant described “*The subject is mentally*. *I saw that the subject is more psychological than it is physical*. *I mean*, *the*, *my mental status was different*, *and you know that the topic (sex) is all based on your psychological status*. *I mean*, *when you are mentally well and there is not too many thing occupying your thinking then you will think about this topic (sex)*. *Otherwise*, *this stuff will be you last priority”* (Participant 5). Thus, concluding that sexual desire was lacking due to mental wellbeing and altered priorities.

### 2. Need for a therapeutic/intimate communication

#### 2.1 Need for information about the effects of cancer on sexual relationships

Receiving information on the effects of cancer and cancer treatment on sexual relationships was felt to be lacking by participants in this study. Information provision during medical consultations was non-existence for some participants, stating *“Zero*. *I did not have any information”* (Participant 2) and “*Honestly*, *I mean*, *at the beginning my information about the disease was not more than 10% of the information that I know about it now*. *And my information about marital relationship (sex relationship) after illness was zero”* (Participant 4). While others experience was that even though they lacked information, their mind was not focused on sexual intimacy, “*As a person*, *I did not have enough information and I also did not try to search for it because it simply did not concern me at the beginning”* (Participant 3).

#### 2.2 Building a rapport is vital for openness

Building a rapport with patients can be difficult, however displaying empathy and ensuring the patient believes they have a trusting relationship, are essential for establishing an interpersonal patient-doctor relationship. One participant felt “*The doctor should breaks the barrier between him and his patient*, *whether by joking*, *whether by joking with anything that is possible to give him the information*, *he can get his joke and tell him a piece of information*. *Possibly this information is very important*. *This is a communication skill*. *I mean*, *the doctor must have the skill of how to deal with his patient”* (Participant 4), demonstrating how important it is for the doctor to have the appropriate communication skills to build rapport. Feeling like a person rather than a disease or number would also promote openness as one participant says “*The doctor’s way of communication will comfort*. *Doctor who is in a hurry and his focused on seeing you quickly*. *They look at you as a patient and he is normally see too many patients”* (Participant 6), suggesting that patients need the doctor to build a rapport with them.

Participants would prefer the doctor to show empathy “*When the patient comes to you*, *you must talk to him as if he is your close friend*, *and you must talk to him in the atmosphere of humor and the atmosphere of friends in order for the patient forgets some of his trauma and to be open for discussion*. *I mean*, *the conversation is from the heart to the heart”* (Participant 2).

Providing a supportive environment is part of making the patient feel as ease to discuss their sexual issues. Participants in this study would like the doctor to make a joke to initiate the conversation about sex and sexual relationships, *“Your patient is your integrity*, *and you have to make sure to look after him in these matters (sexuality)*. *There is no shame*, *he is a man like you*. *I mean*, *if the doctor start the topic with a joke*, *he will give patient a nice atmosphere*, *and if he advises him*, *I mean*, *I will advise every doctor to start the conversation on this topic”* (Participant 1).

#### 2.3 It is a psychological issue, not the cancer

When approaching their doctors regarding sexual issues some participants were advised to consult a psychologist/psychiatrist. The information that participants received from their doctors was that there was no association between cancer and sexual functioning. One participant reports “*The doctor’s answer was that there is no relationship between this disease and this topic (sex life)*. *It’s your psyche (your mental status)*, *you’re thinking about your illness*, *you’re thinking is affecting your relationship*, *all of this reflected on your performance”* (Participant 4). Seeking answers from the doctor relating to sexual performance and sex life resulted in a negative response for some participants, “*In fact*, *I asked him why this happened with me at the beginning (I mean how cancer affected my sexual life)*. *I asked him about the reason for the apathy and he advised me to go to a psychiatrist*, *because the problem is first with me”* (Participant 3), suggesting that sexual relationships are not affected by cancer and it was an individual problem that required psychological intervention. However, this demonstrates a lack of support and understanding from the physician.

#### 2.4 The need for sexual counseling as part of the cancer care

Provide patients with cancer information and knowledge of the sexual issues that can occur is imperative to ensure they receive all the necessary information. Many of the participants reported receiving very little in the way of education related to sexual issues and relationships, however felt that it required greater priority. One participant says “*the awareness (education) section on this topic (sexual issues) is too weak*. *I remember the chemo doctor at beginning*, *he told me that this treatment will affect your emotional life a little*. *I mean two words*, *and he walked on the subject like lightning*. *He never explained anything to m*e” (Participant 2). Being provide with information and education on sexual issues needs to be an essential component of cancer care.

Despite patients with cancer initially wanting to focus on the issues directly related to cancer care and treatment, most patients felt that education on sexual issues was substantially lacking. One participant stated, “*But the chemo doctor told me pen tips that this treatment would affect your sexual life*, *but I*, *of course*, *did not care because it was the beginning of the disease and the first chemotherapy session I was not thinking about the topic at all”* (Participant 2). Patients need to be reassured by the doctors that sexual issues are part of the cancer journey and are important, “*I think*, *the issue is that people think this is not important*. *I mean that the doctor and the treating doctor are concerned about the disease*, *the treatment*, *the side effects*, *but they don’t focus on the patient sex life”* (Participant 4). Shifting priorities so that sexual relationships are discussed is necessary for patients, with one participant commenting “*The second aspect is that the doctor primary responsibility for treating the same problem*, *which is the cancer*, *and he will not focus on patient sex relationship”* (Participant 5).

### 3. Variations in sexual life and sexual experiences

#### 3.1 Sources of information regarding the impacts of cancer on sex life

Initially, patients with cancer-focused on diagnosis and treatment options but gradually raised concerns about the effect of cancer on their sex life. Many participants report not feeling about discussing these personal and private issues with their doctors and used various other methods to obtain their information. One participant reports, “*At the beginning*, *I didn’t discuss this thing (sex life)*, *but I tried to go online and see it on*

*the social media websites”* (Participant 6), his source of information was through social media sites. Others required to look at Arabic specific websites for further information on sexual issues, “*Especially the websites that write in Arabic because my English is not strong enough for me to read full articles”* (Participant 3). Despite many participants reporting that the doctor provided limited information, some felt that this was the most reliable and relatable information “*Doctor said in a funny way*, *that if Fifi abdo (Arabic beautiful dancer) will dance in front of you*, *you will not feel anything (sexual feeling)”* (Participant 7).

#### 3.2 Effects on the partner

Partners can offer support during cancer treatment and provide stability and reassurance that sex life is not the most important issue during the cancer journey. One participant in this study elaborated on his experience with his partner saying “*She stand with me and my side (support me)*, *and she said that don’t worry about that thing (having sex) the most important thing is your health”* (Participant 1). In contrast, another participant reported that a cancer diagnosis had a negative impact on his partner and sexual relationship, “*So after I diagnosed with the disease*, *I was trying to get closer to her*, *but the relationship fell apart on her side*. *I mean*, *she is the one who moved away”* (Participant 3). There were also misconceptions regarding cancer by the partner, which had an effect on the sexual relationship “*Psychologically*, *she is no longer accepts the subject because she has come to feel that it might become an infection*… *Personally*, *I am convinced that it is impossible for it to become an infection*. *But my wife was not very convinced that it could become an infection from this topic (cancer)*” (Participant 3).

#### 3.3 Sexual relationships prior to cancer

The sexual relationships described by the participants prior to a cancer diagnosis were ‘normal’, without any issues. Many reported have a good marital relationship, with one participant commenting “*I mean*, *at the beginning*, *I was a normal person with what I wanted to do*, *and I was physically practicing the marital relationship well and comfortably”* (Participant 6). Similarly, another participant reported a positive experience before cancer saying *“My wife and I lived our life very naturally*, *because sexual life is a very*, *very important part of any couple’s life”* (Participant 2). Others also reported a comfortable and normal sex life saying *“I was living with my wife*, *an excellent marital life*, *psychological comfort*, *and peace”* (Participant 7) and “*The relationship was good*, *I mean*, *similar to other people*. *We was living and happy*, *and there was no problem or anything affecting us”* (Participant 5).

## Discussion

Overall, this study revealed that there is a substantial psychological impact of cancer on sexual relationships among Arab Jordanian cancer survivors. The cultural and societal norms of providing an insight into their marital relationships, including sex life, was highlighted by participants as a taboo topic, and thus preventing any truthful discussions about their sexual needs with their treating team or partners. This is congruent with the study of Sbitti et al where sexual relationships were described as a taboo subject among Arab population groups [18]. This lead to underreporting in research examining unmet sexual needs, as well as limited evidence in the literature on the topic [8].

Being labelled as ‘sexually disabled’ was also emphasized by participants as a barrier to discussing their sexual relationships. In Arabic Jordanian society males are dominant and any discussions about sexual relationships can lead to being labelled as sexually disabled, thus affecting their social position [19]. Hence, Arab males avoid discussions about sexual relationships or need to maintain an acceptable social status in their community [20]. This is aligned with the studies by Alananzeh et al and Butow et al. who reported a low level of sexuality unmet needs which may reflect the taboo nature of discussing sexual needs [8, 9].

In this study, the cancer survivor’s physical condition increased their sense of powerlessness and loss of control over sexual relationships. The physical condition including erectile dysfunction, anejaculation, and changes in sexual performance are well-known side effects of cancer and its active treatment [21]. The sense of powerlessness and loss of control over sexual relationships identified in this study is consistent with the literature where a large proportion of patients reported that they had not been sexually active within the previous month had a desire for some degree of sexual intimacy and that their physical condition or treatment had impaired their sex life [22].

On the other hand, sexual desire was also linked by some participants to their mental wellbeing rather than physical ability. Although participants revealed good or normal sexual relationships before being diagnosed with cancer, they reported a lack of desire for continuing sexual relationships after being diagnosed, which is suggested to be related to mental stability and alteration in priorities. This is also aligned with a systematic review that found the cancer disease and treatment can increase the cancer survivor’s stress and emotional distress which may impact his sexual life. In addition, depression and anxiety which are the main common concerns for cancer survivors may further contribute to sexual problems [23].

The results of this study have demonstrated a major communication gap between participants and their physicians, resulting in lack of support and information resources on the effect of cancer and its treatment. The lack of communication issue has also been reported in the literature and has been identified as one of the main barriers in seeking help and treating sexual problems among cancer survivors [24]. The lack of communication remains problematic as sexual life is considered a high priority by the survivors however is commonly overlooked by physicians. A possible explanation of this gap could be the cultural beliefs, the lack of a trusting physician-patient relationship, lack of appropriate training for the physician, and not feeling comfortable along with embarrassment to discuss such a sensitive topic [25, 26]. A recent systematic review found that the sexual concerns of cancer survivors remain concealed with many cancer survivors not receiving adequate support or information. Findings from this review suggest greater efforts are particularly needed in increasing the assessment and management of treatment-related sexual concerns [27].

Of particular concern in this study was the lack of communication with physicians leading cancer survivors to seek alternative methods to obtain information about the impact of cancer and its treatment on their sexual life, including Arabic specific websites and social media sites. These findings are in line with Alananzeh study which found that Arab cancer survivors obtained information most frequently from the internet (45.5%), general practitioners (39.2%) and telephone information services (21%) [8]. Education related to the sexual relationship and issues as an essential component of their cancer care, was reported to be commonly overlooked by participants’ physicians [6].

The importance of the partner’s support in maintaining a good sexual relationship during cancer and its treatment was emphasized in this study. Many studies highlighted the impact of partner support on the cancer survivors’ psychological distress and improving the survivors QOL [28–30]. However, participants revealed that partners’ misconceptions regarding the impact of cancer on a sexual relationship, in turn, affected their relationships. Some Arab still have several misconceptions regarding cancer including that cancer is incurable, contagious, pre-destined and a source of shame [30, 31]. Perhaps this correlates with a lack of information and resources for both cancer survivors and their partners and absence of engagement of partners in the cancer care journey. This again can be linked to the cultural barrier and sensitivity of sexual related topics.

## Conclusion

This study explored the sexual experience among Jordanian cancer survivors and challenges they face during their cancer journey. Cancer survivors in this study experienced changes in their sexual life due to the impact of cancer and it active treatment, which affect their mental and psychological health. Moreover, it was increasingly apparent that Jordanian cancer survivor’s faces additional challenges in discussing their sexual needs and seeking help from their healthcare providers due to the shame associated with this topic and feeling powerless to take control over their sexual relationships. Interestingly, there appeared to be a clear need for physician to broaden their focus from providing cancer treatment to address patient’s sexual changes and information needs. Subsequently, the study results indicated a crucial need for physicians to encourage patients to disclose their sexual experience in order to help them maintain their sexual and mental health while in the fight against cancer. On the other hand, cancer showed a similar impact on patient’s partners including misconceptions regarding the impact of cancer on sexual relationship and their ability to provide support during the cancer journey. Overall, findings of this study are inevitable for future health services for this specific community and addresses the knowledge gap about sexual needs and experience of Arab people.

### Strength and limitations

Cancer survivors’ sexual needs are one of the neglected issues by Jordanian cancer care providers, including nurses and physicians, due to cultural and social perceptions. The main strength of the study is that it is the first to use a qualitative approach to explore sexuality experience of Jordanian cancer survivors by understanding the sexual needs of this cultural group and connect these needs with their cultural beliefs and practices. Another strength of the study was the rigour in which it was conducted.

The interviews were conducted by a skilled Arab researcher and nurse who ensured that the study participants were comfortable talking about their sexual needs and handled unexpected reactions appropriately. This led to participants freely sharing their experience and expressing their sexual needs, which was the aim of the study.

Although this study was conducted using rigorous methods, certain limitations inherent in undertaking such studies needs to be acknowledged. Firstly, the respondents in this study were seven Arab men hence the results cannot be extrapolated to Arab women who may have different sexual needs. Secondly, this study was limited to long term cancer survivors and it is well known that the experience of newly diagnosed patients is different from long-term survivors. Hence the findings may not apply to all cancer patients. In addition, the small sample size does not support generalization to the broad and diverse population of Arab cancer survivors. There are also biases introduced using the snowballing sampling as a method of recruitment such as selection bias which is a limitation of this study.

### Clinical implications

#### Implication for practice

The study indicated a crucial need for physicians to encourage patients to disclose their sexual experience in order to help them maintain their sexual and mental health while in the fight against cancer. The physician should initiate the conversation and encourage patients to be open about their sexual needs. The nurses and allied health professionals can also be engaged in the conversation with patients by taking an active role in the discussion. Such conversation will assist in closing the knowledge gap about the effect of cancer on the sexual activity of the patients. Finally, the family and partners of the patients should also be approached and engaged by health professionals in the discussion to address their needs or sexual issues.

### Implications for further research

Further research is needed to explore the sexual needs of female Arab cancer survivors as well their care providers. Given the sensitive nature of the topic, it is important that female cancer survivors are approached by female researchers to identify their sexual needs so that gender-specific strategy can be developed and implemented. Further research is also needed to explore the views of the physicians to provide sexual counselling for cancer survivors.

## References

[1] World Health Organization. Cancer: Overview. World Health Organization. https://www.who.int/health-topics/cancer#tab=tab_1

[2] Pan American Health Organization. Cancer Profile 2020. Pan American Health Organization. https://www.paho.org/hq/index.php?option=com_docman&view=download&category_slug=4-cancer-country-profiles-2020&alias=51561-global-cancer-profile2020&Itemid=270&lang=fr

[3] King Hussein Cancer Foundation. Cancer Local Statistics in Jordan 2011. http://www.khcc.jo/en/section/local-statistics

[4] The Jordanian Ministry of Health. Cancer Incidence in Jordan—2016. Jordan Cancer Registry: Non-Communicable Disease Directorate. The Jordanian Ministry of Health.

[5] Hawkins Y, Ussher J, Gilbert E, Perz J, Sandoval M, Sundquist K. Changes in sexuality and intimacy after the diagnosis and treatment of cancer: the experience of partners in a sexual relationship with a person with cancer. *Cancer Nurs*. 2009 Jul-Aug;32(4):271–80. doi: 10.1097/NCC.0b013e31819b5a93 . 19444088

[6] Twitchell D, Wittmann D, Hotaling J, Pastuszak A. Psychological Impacts of Male Sexual Dysfunction in Pelvic Cancer Survivorship. *Journal of Sexual Medicine Review*. 2019;7(4):614–626 10.1016/j.sxmr.2019.02.003 PMC6763375 30926459

[7] Olsson C, Berglund A-L, Larsson M, Athlin E. Patient’s sexuality–A neglected area of cancer nursing? *European Journal of Oncology Nursing*. 2012;16(4):426–431. doi: 10.1016/j.ejon.2011.10.003 22036773

[8] Alananzeh IM, Levesque JV, Kwok C, Salamonson Y, Everett B. The Unmet Supportive Care Needs of Arab Australian and Arab Jordanian Cancer Survivors: An International Comparative Survey. *Cancer Nursing*. 2019;(3):51. doi: 10.1097/NCC.0000000000000609 29757770

[9] Butow PN, Bell ML, Aldridge LJ, et al. Unmet needs in immigrant cancer survivors: a cross-sectional population-based study. *Supportive Care in Cancer*. 2013;21(9):2509–2520. doi: 10.1007/s00520-013-1819-2 23625019

[10] Saleh M, Barlow-Stewart K, Meiser B, Tucker K, Eisenbruch M, Kirk J. Knowledge, attitudes and beliefs of Arabic-Australians concerning cancer. Psychooncology 2012; 21: 195–202. doi: 10.1002/pon.1884 22271540

[11] Baron-Epel O, Granot M, Badarna S, Avrami S. Perceptions of breast cancer among Arab Israeli women. Women Health 2004; 40: 101–16. doi: 10.1300/J013v40n02_07 15778141

[12] Merry J, Robinson J. Review—CALD cancer information and support groups in South Western Sydney Local Health District. (2014).

[13] Tong A, Sainsbury P, Craig J. Consolidated Criteria for Reporting Qualitative Research (COREQ): A 32-Item Checklist for Interviews and Focus Groups. International Journal for Quality in Health Care 19(6):349–57 doi: 10.1093/intqhc/mzm042 17872937

[14] Casado BL, Negi NJ, Hong M. Culturally Competent Social Work Research: Methodological Considerations for Research with Language Minorities. *Social Work*. 2012;57(1):1–10. doi: 10.1093/sw/swr002 22768624

[15] Braun V, Clarke V. Using thematic analysis in psychology. *Qual Res Psycho*. 2008;3(2):77–101.

[16] Eltaiba N. Counseling with Muslim Refugees: Building Rapport. *Journal of Social Work Practice*. 2014;28(4):397–403. doi: 10.1080/02650533.2013.875523

[17] Graneheim U, Lundman B. Qualitative content analysis in nursing research: concepts, procedures and measures to achieve trustworthiness. Nurse education today. 2004:24(2):105–112. doi: 10.1016/j.nedt.2003.10.001 14769454

[18] Sbitti Y, Kadiri H, Essaidi I, et al. Breast cancer treatment and sexual dysfunction: Moroccan women’s perception. *BMC Women’s Health*. 2011;11(1):29–33. doi: 10.1186/1472-6874-11-29 21668971 PMC3143933

[19] Abboud S., Lanier Y., Jemmott L., & Sommers M. (2019). Navigating Virginities: Enactment of Sexual Agency among Arab Women in the USA. *Culture Health &* *Science*. 2019;21(1):1–14. 10.1080/13691058.2018.1539249 30646837

[20] Al-Krenawi A, Jackson S. Arab American Marriage: Culture, Tradition, Religion, and the Social Worker. *Journal of Human Behavior in the Social Environment*. 2014;24(2):115–137. doi: 10.1080/10911359.2014.848679

[21] Voznesensky I, Shaw E, DeLay K, Yafi F. Begnine Prostatic Hyperplasia Treatment Options and their Effects on Sexual Function. *Sexual Medicine Review*. 2016; 5(1): 87–102.

[22] Bond CB, Jensen PT, Groenvold M, Johnsen AT. Prevalence and possible predictors of sexual dysfunction and self-reported needs related to the sexual life of advanced cancer patients. *Acta Oncologica*. 2019;58(5):769–775. doi: 10.1080/0284186X.2019.1566774 30724646

[23] Maiorino M I, Chiodini P, Bellastella G, Giugliano D, Esposito K. Sexual dysfunction in women with cancer: a systematic review with meta-analysis of studies using the Female Sexual Function Index. *Endocrine*. 2016;54(2):329–341. doi: 10.1007/s12020-015-0812-6 26643312

[24] Abahssain H, Lalya I, M’Rabet F zahra EL, et al. Breast Cancer in Moroccan Young Women: A Retrospective Study. *BMC Research Notes*. 2010;3:286–294. doi: 10.1186/1756-0500-3-286 21059204 PMC2992542

[25] Lindau ST, Surawska H, Paice J, Baron SR. Communication about sexuality and intimacy in couples affected by lung cancer and their clinical-care providers. *Psycho- oncology*. 2011;20(2):179–185. doi: 10.1002/pon.1787 20540168 PMC3319754

[26] Vermeer WM, Bakker RM, Kenter GG, Stiggelbout AM, Ter Kuile MM. Cervical cancer survivors’ and partners’ experiences with sexual dysfunction and psychosexual support. *Supportive care in cancer*: *official journal of the Multinational Association of Supportive Care in Cancer*. 2016;24(4):1679–1687. doi: 10.1007/s00520-015-2925-0 26412245 PMC4766206

[27] Spendelow JS, Eli Joubert H, Lee H, Fairhurst BR. Coping and adjustment in men with prostate cancer: a systematic review of qualitative studies. *Journal of Cancer Survivorship*: *Research and Practice*. 2018;12(2):155–168. doi: 10.1007/s11764-017-0654–8. PMC5884891 29063497

[28] Reese J, Beach M, Smith K, et al. Effective patient-provider communication about sexual concerns in breast cancer: a qualitative study. *Supportive Care in Cancer*. 2017;25(10):3199–3207. doi: 10.1007/s00520-017-3729-1 28451911 PMC5803445

[29] Kamen C, Jabson JM, Mustian KM, Boehmer U. Minority stress, psychosocial resources, and psychological distress among sexual minority breast cancer survivors. *Health Psychology*. 2017;36(6):529–537. Accessed December 12, 2020.https://search.ebscohost.com/login.aspx?direct=true&AuthType=shib&db=edo&AN=123284536&site=eds-live doi: 10.1037/hea0000465 28165265 PMC5444950

[30] Rini C, Redd WH, Austin J, et al. Effectiveness of Partner Social Support Predicts Enduring Psychological Distress After Hematopoietic Stem Cell Transplantation. *Journal of Consulting &* *Clinical Psychology*. 2011;79(1):64–74. 10.1037/a0022199 PMC3690958 21261435

[31] Scott N, Donato HC, Crane M, et al. Knowledge, attitudes and beliefs about lung cancer in three culturally and linguistically diverse communities living in Australia: a qualitative study. *Health Promotion Journal of Australia*. 2014;25(1):46–51. doi: 10.1071/HE13095 24739779

